# A down-shifting Eu^3+^-doped Y_2_WO_6_/TiO_2_ photoelectrode for improved light harvesting in dye-sensitized solar cells

**DOI:** 10.1098/rsos.171054

**Published:** 2018-02-07

**Authors:** J. Llanos, I. Brito, D. Espinoza, Ramkumar Sekar, P. Manidurai

**Affiliations:** 1 Department of Chemistry, Universidad Católica del Norte, Avda. Angamos 0610, Antofagasta, Chile; 2 Department of Chemistry, Universidad de Antofagasta, Avda. Angamos 0601, Antofagasta, Chile; 3 Department of Chemistry, Universidad de Chile, Las Palmeras 3425, Santiago, Chile; 4 Department of Physics, Universidad de Concepcion, Barrio Universitario, Casilla 160-C, Concepción, Chile

**Keywords:** dye-sensitized solar cells, down-shifting, photoelectrode, lanthanide ion

## Abstract

Y_1.86_Eu_0.14_WO_6_ phosphors were prepared using a solid-state reaction method. Their optical properties were analysed, and they was mixed with TiO_2_, sintered, and used as a photoelectrode (PE) in dye-sensitized solar cells (DSSCs). The as-prepared photoelectrode was characterized by photoluminescence spectroscopy, diffuse reflectance, electrochemical impedance spectroscopy (EIS) and X-ray diffraction. The photoelectric conversion efficiency of the DSSC with TiO_2_:Y_1.86_Eu_0.14_WO_6_ (100:2.5) was 25.8% higher than that of a DSCC using pure TiO_2_ as PE. This high efficiency is due to the ability of the luminescent material to convert ultraviolet radiation from the sun to visible radiation, thus improving the solar light harvesting of the DSSC.

## 1. Introduction

Dye-sensitized solar cells (DSSCs) are part of this third generation, and they have attracted considerable attention because of their low cost, easy fabrication and relatively high conversion efficiencies [1,2]. DSSCs were proposed by O'Regan and Grätzel in 1991 [3]. However, the poor response of DSSCs to red and near-infrared (NIR) light has been a significant impediment for achieving higher photocurrents and efficiencies. Therefore, to develop competitive DSSC technology, further increasing their efficiency by widening their operational spectral range is necessary.

The spectral response of conventional DSSCs is very narrow compared to the whole wavelength range of sunlight. The band between 400 and 700 nm only accounts for approximately 43% of the sun's total radiant energy [4]. To harvest energy in the red and NIR regions of the solar spectrum, new types of dyes, quantum dots and co-sensitizers are currently under development [5–8]. All of these routes still suffer from some disadvantages, however, such as: (i) the new dyes do not absorb at wavelengths above 750 nm, i.e. in the red region; (ii) organic dyes decompose relatively easily upon interacting with the ${{\text{I}}_{3}}^{-}$ electrolyte; and (iii) quantum dots are sensitive to the presence of moisture or oxygen and have restrictions for their use in sensitized solar cells [9,10]. A novel way to enhance the conversion efficiency in DSSCs is the use of down-shifting (DS), down-conversion (DC) and up-conversion (UC) luminescent materials as components of the solar cells. Generally, DS, DC and UC materials consist of rare earth (RE)-doped inorganic host materials. The intra-*4f* transitions in RE-doped luminescent materials can be efficiently used as either NIR-to-visible up-convertors or ultraviolet (UV)-to-visible down-convertors for applications in DSSCs.

On the other hand, the literature reports that exposure to UV light produces serious damage to the DSSC since the iodine present in the electrolyte is irreversibility consume under UV light [11]. Down-conversion or down-shifting phosphors are suitable materials for reducing this disadvantage in dye-sensitized solar cells. Recently, Zhang *et al*. described how the use of the inorganic phosphors LAVO4:Dy3+ introduced into a DSSC, not only improves the electrical conversion efficiency (*ca* 23%), but also expands considerably the lifetime of the solar cell [12].

This paper is part of our continuing research on the synthesis, applications, characterization and luminescent properties of inorganic phosphors containing rare-earth cations [13–16]. In this report, we focused on the preparation of Eu^3+^-doped Y_2_W0_6_ and its application in photoelectrodes (PE) in DSSCs in order to investigate the possibility of increasing the spectral response of DSSCS using down-shifting luminescent materials.

## 2. Experimental

### 2.1. Materials

All chemical reagents including Eu_2_O_3_, Y_2_O_3_, WO_3_, TiO_2_ nanopowder (21 nm), I_2_, LiI, tetrabutylammonium iodide, and t-octylphenoxypolyethoxyethanol as emulsification agent (Triton X-100) were analytical grade and supplied by Sigma-Aldrich. Conducting glass plates of indium tin oxide (ITO) with a surface resistivity of 8–12 Ω sq^−1^ and the sensitizing dye N-719 (RuL_2_(NCS)_2_:2TBA (L = 2,2′-bipyridyl-4,4′-dicarboxylic acid)) were also purchased from Sigma-Aldrich and used without further treatment.

### 2.2. Preparation of Eu^3+^-doped Y_2_W0_6_ nanoparticles

The Y_2_WO_6_:Eu^3+^ phase was prepared via a solid-state reaction at high temperature [17,18]. All phosphors were synthesized from a thoroughly ground mixture of the corresponding oxides, Y_2_O_3_, Eu_2_O_3_ and WO_3_, in stoichiometric proportions. The mixtures were placed in an alumina boat and heated to 973 K for 10 h before cooling below 400 K. The sample was removed from the crucible, ground into a powder, and reheated for 10 h at 1273 K. This procedure was repeated, and the sample was finally heated to 1373 K for another 10 h. All of these synthetic processes were performed under an air atmosphere. Optical inspection of the products showed homogeneous white powders.

Powder X-ray diffraction (PXRD) data were collected using a Bruker D8 Advance diffractometer fitted with a graphite monochromator using Cu K*α* radiation (*λ* = 1.54057 Å) across the range of 10° ≤ 2*θ* ≤ 60° to determine the phase purity. The experimental PXRD patterns for the doped and un-doped samples were almost in perfect agreement with those reported in the inorganic crystal structure database (ICSD) database [19].

### 2.3. Preparation of dye-sensitized solar cell

Different amounts of Y_1.86_Eu_0.14_WO_6_ (1%, 2%, 2.5% and 3%) were dispersed in a TiO_2_ sol, ultrasonicated, stirred for about 30 min and finally used as slurry. The PE was prepared by the doctor-blade technique [20,21]. After air-drying, the electrodes were sintered at 450°C for 30 min and soaked in an N-719 dye solution (3 × 10^−4^ M in ethanol) for 24 h. For comparison, a PE without Y_1.86_Eu_0.14_WO_6_ (i.e. pure TiO_2_) was also prepared. Finally, the DSSC was assembled by filling an electrolyte solution (0.60 M tetrabutylammonium iodide, 0.05 M I_2_, and 0.10 M LiI in acetonitrile) between the dye-sensitized Y_1.86_Eu_0.14_WO_6_/TiO_2_ electrode and a platinized conducting glass electrode. Both electrodes were clipped together, and a cyanoacrylate adhesive was used as a sealant to prevent the electrolyte solution from leaking. For comparison, a DSSC was prepared with the pure TiO_2_ PE using the same method.

### 2.4. Characterization

The phase purities were determined via PXRD analysis (vide supra). The nanocrystalline Y_1.86_Eu_0.14_WO_6_ phosphor surface morphologies were determined via scanning electron microscopy (SEM, JEOL, JSM-6360LV). The photoluminescence (PL) spectra were measured using a JASCO FP-6500 spectrofluorometer. All of the spectra were collected at room temperature. The sample quantities were identical across all experiments so that the photoluminescence intensities could be compared. A Perkin-Elmer Lambda 20 UV-Vis spectrophotometer equipped with a Labsphere RSA-PE-20 diffuse reflectance accessory was used to measure the diffuse reflectance of the samples over the range of 200--600 nm (6.2--2.1 eV). The photovoltaic test of the DSSCs was performed using a PET cell tester (model CT80AAA, Photo Emission Tech., Inc. USA), under simulated AM 1.5 solar illumination of one sun (100 mW cm^–2^) from a 300 W Xe lamp. The intensity was adjusted using a calibrated c-Si solar cell. The I–V measurements were attained using a Keithley Sourcemeter model 2400. The electrochemical impedance spectroscopy (EIS) analysis were carried out with Solartron 1280 impedance analyser. Nyquist plots were measured at open circuit voltage (*V*_oc_) over a frequency range of 1–10^4^ under one-sun illumination. To determine the amount of dye desorbed from the photoelectrode a 1 M solution of NaOH was used and the UV-Vis spectra were recorded in a Perkin-Elmer Lambda 25 spectrophotometer.

## 3. Results and discussion

**Figure 1.**
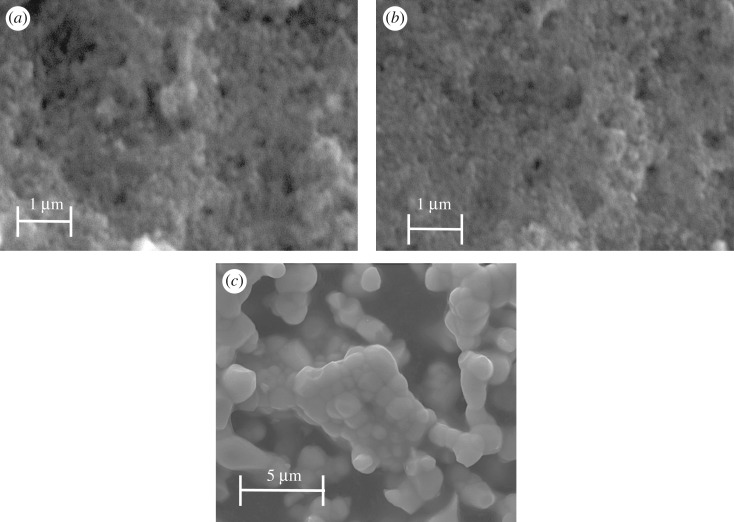
SEM images of (*a*) TiO_2_, (*b*) TiO_2_/Y_1.86_Eu_0.14_WO_6_ (100:2.5) and (*c*) Y_1.86_Eu_0.14_WO_6_.

The crystallite morphology of Y_1.86_Eu_0.14_WO_6_ was inspected via SEM. The results revealed that the samples are spherical and exhibit some tendency to aggregate, as shown in figure 1 [18]. The morphology of the layers was also inspected by SEM. Figure 1 shows the typical SEM images of bare TiO_2_ and the doped samples. No remarkable differences were observed. The phosphor was embedded within the structure of TiO_2_, which is an important characteristic to obtain a good dye distribution [22].

**Figure 2.**
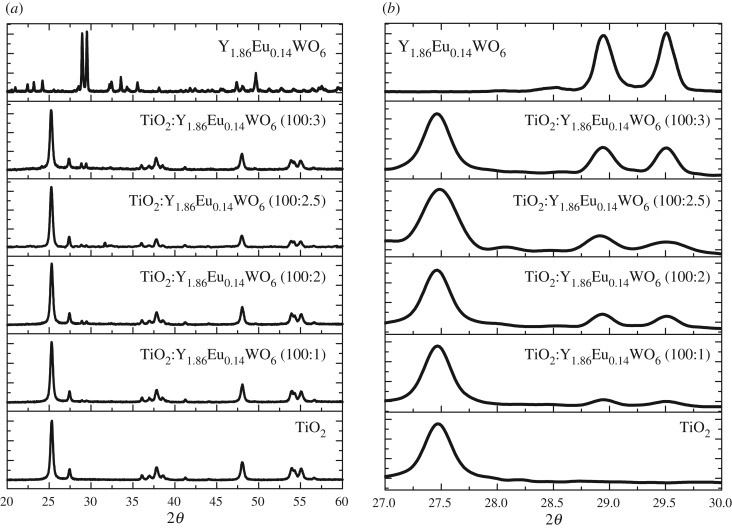
(*a*) XRD patterns of TiO_2_ mixed with different content of Y_1.86_Eu_0.14_WO_6_. (*b*) XRD patterns in the 2*θ* region of 27--30°.

Additionally, the Scherrer equation (*D* = 0.90*λ*/*β*cos*θ*) can estimate the crystallite size of the luminescent material from the PXRD results if the crystals are smaller than 1000 Å. In this equation, *D* is the average grain size, *λ* is the wavelength of the radiation used in the diffraction experiments (vide supra), *θ* is the diffraction angle and *β* is the full width at half maximum (FWHM) of the observed peak [23,24]. Since small angular differences are associated with large spatial distances (inverse space), the broadening of a diffraction peak is expected to reflect some scaling feature in the crystal. The strongest diffraction peak located at 2*θ* = 29.5° was used to calculate the grain size of Y_1.86_Eu_0.14_WO_6_. Our results indicate that the crystallite size for the sample is approximately 62 nm. The PXRD patterns of pure TiO_2_ and Y_1.86_Eu_0.14_WO_6_ (1%, 2%, 2.5% and 3% by weight) dispersed in TiO_2_ and pure Y_1.86_Eu_0.14_WO_6_ are displayed in figure 2.

**Figure 3.**
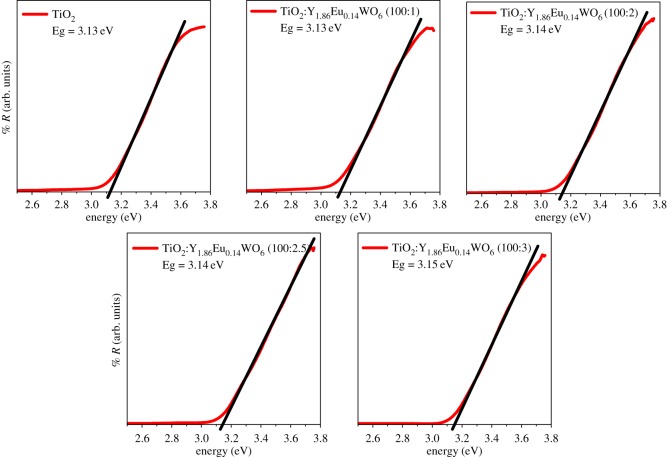
UV-visible diffuse reflectance curves of TiO_2_ mixed with different content of Y_1.86_Eu_0.14_WO_6_. The band gaps of pristine TiO_2_ and TiO_2_ with different contents of phosphors were calculated by the Tauc's plot method.

The corresponding UV-Vis diffuse reflectance spectra of the pure TiO_2_ and the corresponding Y_1.86_Eu_0.14_WO_6_ (1%, 2%, 2.5% and 3% in weight) dispersed in TiO_2_ are superimposed in the figure 3. The band edges are analogous and indicate a band gap energy between 3.13 and 3.15 eV. The commonly reported value for the energy band gap of TiO_2_ is 3.2 eV [25]. Ground state diffuse reflectance absorption studies for the as-prepared photoelectrodes show that semiconductor properties of the TiO_2_ were not obstructed by the inorganic phosphor.

**Figure 4.**
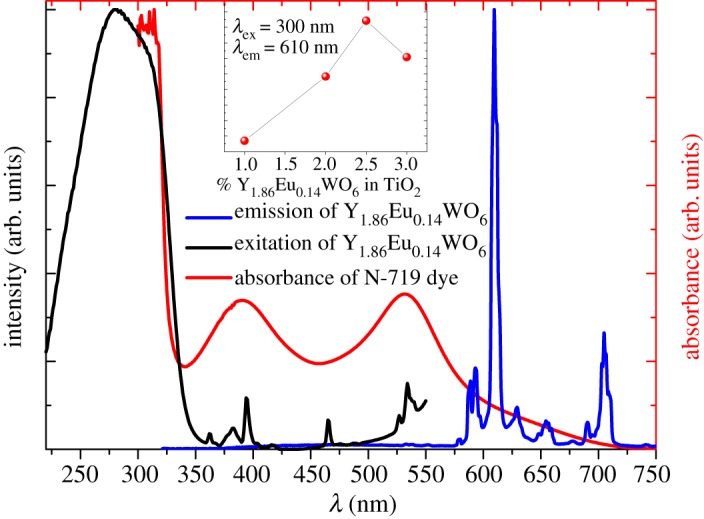
Excitation and emission spectra of Y_1.86_Eu_0.14_WO_6_ compared with the absorbance of N-719 dye.

The synthesized phosphor shows the typical excitation and emission bands. The photoluminescence excitation spectrum (PLE) exhibits a broad charge transfer band centred at about 300 nm along with very weak f-f transitions at about 395 nm (^7^F_0_ → ^5^L_6_), 465 nm (^7^F_0_ → ^5^D_2_) and 540 nm (^7^F_0_ → ^5^D_1_). The broad, intense band consists of overlapping O^2−^-Eu^3+^ and O^2−^-W^6+^ charge transfer bands. Upon excitation at 300 nm, the resulting emission spectrum originates from the transition between the ^5^D_0_ excited state to the ^7^F_j_ ground states (*j *= 0, 1, 2, 3, 4) for the 4f^6^ configuration of Eu^3+^, with the most prominent electric dipole transition, ^7^D_0_ → ^5^F_2_, centred at about 610 nm. Figure 4 shows the excitation and emission spectra of Y_1.86_Eu_0.14_WO_6_ compared with the absorbance of N-719 dye.

**Figure 5.**
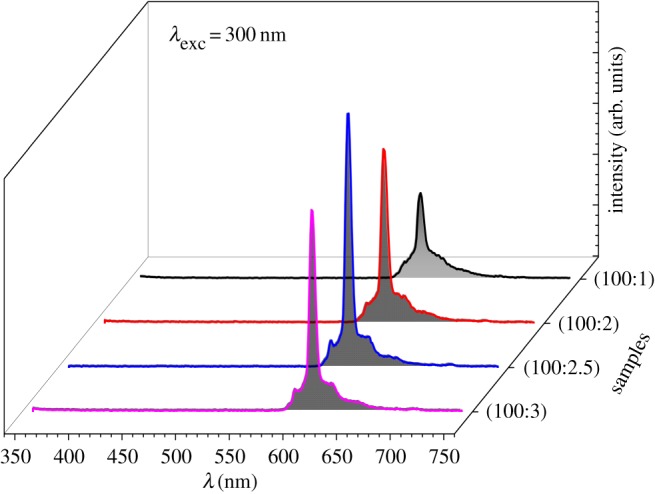
Emission spectra of the film electrodes at wavelength of 300 nm.

Using ITO conductive glass as a substrate, the TiO_2_:Y_1.86_Eu_0.14_WO_6_ photoelectrodes were prepared using the doctor-blade technique [20,21]. The emission spectra of the film electrodes at a wavelength of 300 nm exhibit an intense peak at 610 nm. The effect of the phosphor phase on the emission intensity is shown in figure 5. The emission intensity reaches a maximum with the TiO_2_:Y_1.86_Eu_0.14_WO_6_ (100:2.5) electrode (see inset figure 4). The luminescent material can convert the UV radiation from the sun to visible radiation, thus improving the solar light harvesting of the DSSC.

**Figure 6.**
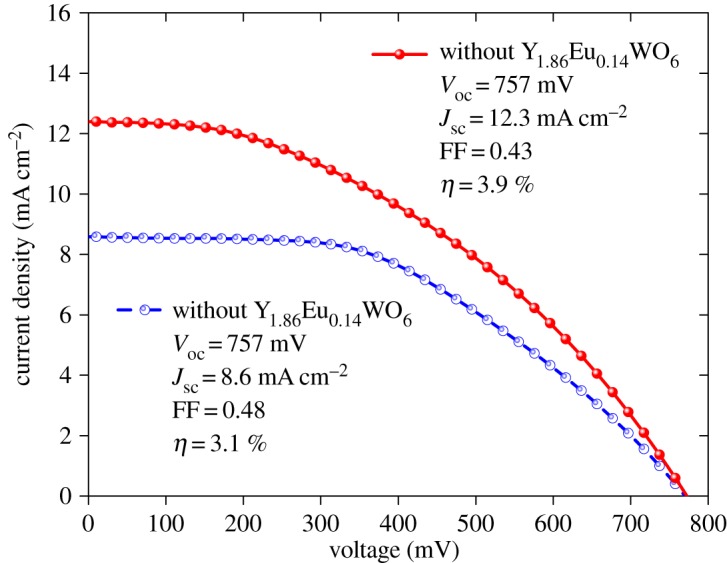
Current density--voltage characteristics for bare and phosphor doped TiO_2_ based DSSCs under illumination of simulated solar light (AM 1.5, 100 mW cm^−2^).

Figure 6 shows the photovoltaic performance of DSSC using the mixed TiO_2_:Y_1.86_Eu_0.14_WO_6_ (100:2.5) and pure TiO_2_ PEs. To obtain significant data, five DSSCs were prepared with and without 2.5 wt% of the luminescent material. When the PE consists of pure TiO_2_, the DSSC has a short-circuit current density (*J*_sc_) of 8.6 mA cm^–2^, an open circuit voltage (*V*_oc_) of 0.76 V, a field-factor (FF) of 0.48 and a photoelectric conversion efficiency (PCE) of 3.1%. The DSSCs with TiO_2_:Y_1.86_Eu_0.14_WO_6_ (100:2.5) photoelectrode show a *J*_sc_ of 12.3 mA cm^–2^, a *V*_oc_ of 0.76 V, a FF of 0.43 and a PCE of 3.9%. The DSSC with the down-shifting material exhibits a higher *J*_sc_ due to the increase in photo-generated photons in the visible region converted from the UV region of the solar spectrum. By the other hand, the Rs and Rsh values for the DSSC using the mixed TiO_2_:Y_1.86_Eu_0.14_WO_6_ (100:2.5) photoelectrode were 9.8 Ω and 1657 Ω, respectively. Whereas for the DSSC using bare TiO_2_ the values were and 10.8 Ω cm^2^ and 2036 Ω cm^2^, respectively.

**Figure 7.**
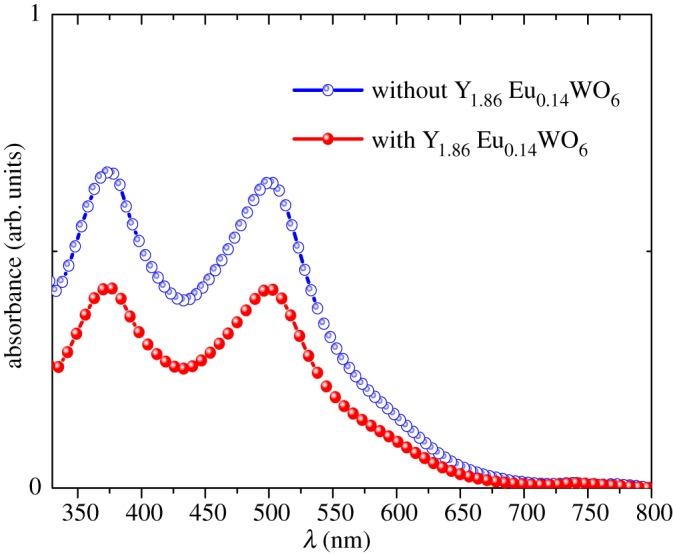
UV-Vis absorbance spectra of the N-719 molecules detached from the photoanodes.

To determine the amount of dye loading in both photoanodes (TiO_2_ and TiO_2_:Y_1.86_Eu_0.14_WO_6_), the photoelectrodes were soaked in a 1 M NAOH solution for 12 h. The absorption spectra of the desorbed dye represented the amount of dye absorbed for the photoanodes. Figure 7 shows the UV-Vis absorbance spectra of the dye molecules detached from both photoelectrodes. The amount of absorbed N-719 was 4.61 × 10^−9^ and 2.94 × 10^−9^ mol cm^−2^ for TiO_2_ and TiO_2_:Y_1.86_Eu_0.14_WO_6_, respectively. Remarkable is the fact that the TiO_2_:Y_1.86_Eu_0.14_WO_6_ (100:2.5) show less absorption than the pristine TiO_2_, their photovoltaic properties are better. The DSSC parameters obtained in our study are shown in table 1.

**Table 1.** Photovoltaic (PV) properties of bare and phosphor doped TiO_2_ based DSSCs.

DSSC	*J*_sc_ (mA cm^−2^)	*V*_oc_ (mV)	FF	PCE (%)	dye (mol cm^−2^)
TiO_2_	8.6	757	0.48	3.1	4.61 × 10^−9^
TiO_2_:Y_1.86_Eu_0.14_WO_6_	12.3	757	0.43	3.9	2.94 × 10^−9^

**Figure 8.**
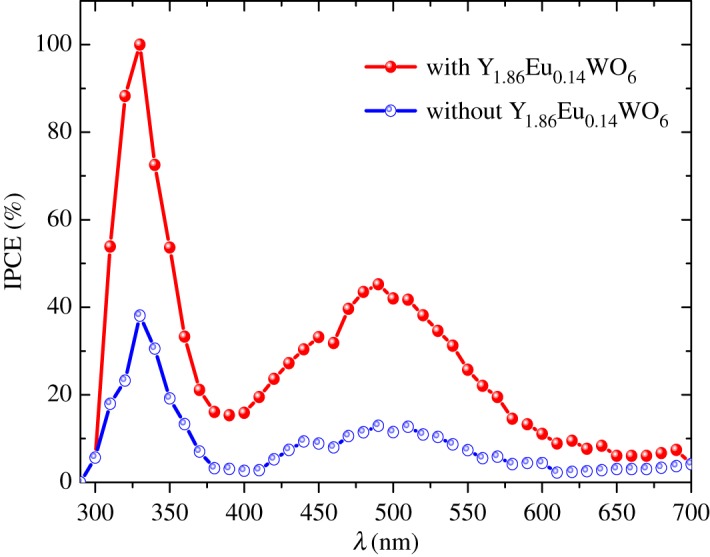
Incident photon to current conversion efficiency (ICPE) curves for bare and phosphor doped TiO_2_ based DSSCs.

The incident photon-to-current efficiency (IPCE) for the dye-sensitized solar cell of DSSC with down-shifting material and with pure TiO_2_ were measured and are shown in figure 8. The IPCE of the dye-sensitized solar cell depends on the incident light harvesting and light scattering [26,27]. The enhancement of the IPCE in the range 300–400 nm can be associated with ^5^D_0_ → ^7^F_1_, ^5^D_0_ → ^7^F_2_ and ^5^D_0_ → ^7^F_4_ transitions of Eu^3+^ under excitation at 300 nm (CTB O^2−^-Eu^3+^). The higher IPCE is attributed to the down-shifting from the ultraviolet light to visible light due to the presence of Y_1.86_Eu_0.14_WO_6_ in the photoelectrode [28]. The ultraviolet light converted to visible was reabsorbed by the N-719 dye causing higher IPCE [29]. Our results are in agreement with those reported in the literature where the DS or DC phosphor layers were directly integrated into the DSSC surface to increase the spectral response of the cell [30].

**Figure 9.**
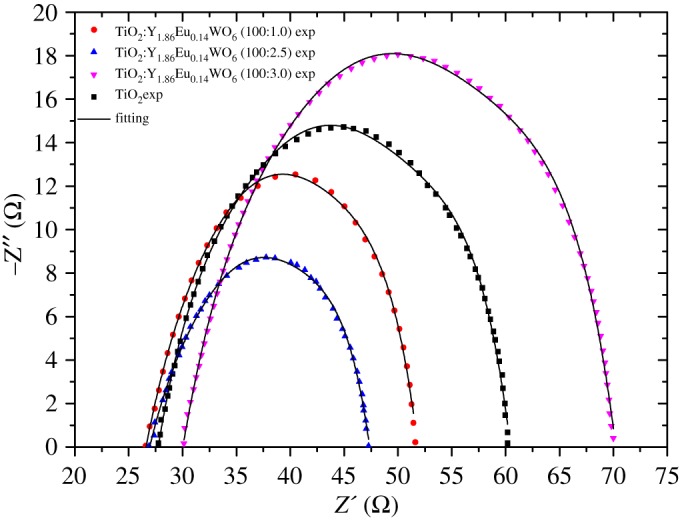
Nyquist plots of the DSSCs based on TiO_2_/Y_1.86_Eu_0.14_WO_6_ (100:0; 100:1; 100:2.5 and 100:3) photoelectrodes measured at *V*_oc_ under one-sun illumination.

On the other hand, the electrochemical impedance spectroscopy (EIS) is an important tool to understand the transport properties within an electrochemical system [31–34]. The EIS spectra of the DSSCs with different photoelectrodes were analysed over a frequency range of 1–10 kHz under one-sun illumination at *V*_oc_. The recorded spectra show a circle at low frequency corresponds to the impedance of TiO_2_/electrolyte interface. Figure 9 shows Nyquist plots of DSSC made with and without luminescent material under one-sun illumination. As shown in figure 9, the smallest circle corresponds to phosphor doped DSSC, which means a decrease in the interfacial resistance and an increase in the efficiency of the cell. This also indicates that the charge transport resistance is lower for the DSSC using the mixed TiO_2_:Y_1.86_Eu_0.14_WO_6_ (100:2.5) (3.6 Ω) than that using the bare TiO_2_ (4.8 Ω). This also indicates less impedance suggesting a faster electron transport, and consequently an improvement in the conversion efficiency. If the concentration of DS phosphor exceeds 2.5%, the charge carrier transportation is hindered. According to Yin *et al*., the TiO_2_:Y_1.86_Eu_0.14_WO_6_ (100:2.5) must have the smaller Rct value. This implies that the electron transfer from the TiO_2_:Y_1.86_Eu_0.14_WO_6_ to the electrolyte is more efficient than that of the pristine TiO_2_ electrode [35].

More work in order to prepare Y_2−x_Eu_x_WO_6_/TiO_2_ core-shell nanoparticles as photoelectrode (PE) for use in dye sensitized solar cells (DSSCs) is in progress.

## 4. Conclusion

The optimized TiO_2_:Y_1.86_Eu_0.14_WO_6_ (100:2.5) mixture was used as a photoelectrode to fabricate DSSCs that were more efficient than DSSCs based on pure TiO_2_ photoactive electrodes. The solar conversion efficiency for a DSSC with the mixed PE reached 3.9%, which is 25.8% higher than that of a DSCC using pure TiO_2_ as the PE.

On the other hand, ground state diffuse reflectance absorption measurements indicated that the addition of the down-shifting phosphor does not affect the semiconductor character of TiO_2_. Finally, in this work we demonstrated that the down-shifted luminescence could be an effective way to improve the sunlight harvesting of solar cells. The last result is supported by electrochemical impedance spectroscopy and optical measurements.

## Data accessibility

General method, materials used and data sheets figures of this article are available at Dryad (http://dx.doi.org/10.5061/dryad.t04k1) [36].
